# Type 5 Diabetes Mellitus: Pathophysiology, Clinical Phenotype, and Nutritional Management

**DOI:** 10.1002/dmrr.70212

**Published:** 2026-07-30

**Authors:** İrem Nur Şahin Anılgan, Aslıhan Atar‐Uğurlu

**Affiliations:** ^1^ Department of Nutrition and Dietetics Faculty of Health Science Istanbul Beykent University Istanbul Turkey

**Keywords:** low body mass index, malnutrition‐related diabetes (MRDM), nutritional rehabilitation, sarcopenia, type 5 diabetes

## Abstract

Type 5 Diabetes Mellitus (T5DM) is an emerging diabetes phenotype linked to chronic undernutrition. It predominantly affects young, lean individuals in low‐ and middle‐income countries. After its formal recognition by the International Diabetes Federation in 2025, T5DM resurfaced as a clinically relevant insulin‐deficient phenotype. It is now viewed as distinct from classical autoimmune and insulin‐resistant forms of diabetes. This review synthesises recent evidence on the epidemiology, pathophysiology, clinical characteristics, and management of T5DM. Recent studies show that T5DM is influenced by early‐life nutritional deprivation. This fits within the Developmental Origins of Health and Disease (DOHaD) framework. Undernutrition during key developmental periods harms pancreatic morphogenesis. It creates a permanent reduction of functional beta‐cell mass through epigenetic programming. Clinically, T5DM often appears before age 30. Patients have low body mass index, sarcopenia, marked insulinopenia, absence of diabetes‐associated autoantibodies, and resistance to ketosis, despite significant hyperglycemia. Diagnostic challenges remain, as patients are frequently misclassified as type 1 or type 2 diabetes. Relying only on body mass index is insufficient. Accurate diagnosis requires a multidimensional assessment including nutritional history, metabolic features, and exclusion of monogenic diabetes. T5DM is a distinct insulin‐deficient phenotype caused by chronic undernutrition. Effective management requires more than glycaemic control. Nutritional rehabilitation is key, with a focus on adequate energy intake, high–biological–value protein supplementation, and correction of key micronutrient deficiencies. Given preserved insulin sensitivity, low‐dose insulin should be used cautiously to avoid iatrogenic hypoglycemia. Better recognition and tailored management strategies are needed for optimal clinical outcomes.

## 1 Introduction

The global diabetes epidemic is more varied than traditional Type 1 (T1DM) and Type 2 (T2DM) classifications suggest [1]. Atypical diabetes phenotypes are common in low and middle‐income countries (LMICs), making current diagnostic and therapeutic guidelines less effective [2]. In this context, ‘Type 5 Diabetes’ (T5DM) has become one of the most discussed and significant new categories in modern diabetology.

A major shift occurred on 8 April 2025, when the International Diabetes Federation (IDF) officially recognised malnutrition‐related diabetes as ‘Type 5 Diabetes’ at the World Diabetes Congress in Bangkok [3]. This move highlights that diabetes is also shaped by food insecurity and early‐life nutritional deficiencies. Thus, it is not just a result of genetic predisposition or obesity‐driven metabolic dysfunction [4]. Including T5DM in the classification is a major step for developing specific diagnostic criteria. It also helps direct research funding to this ‘neglected epidemic’ [5, 6].

The main clinical challenge is that T5DM patients are often misdiagnosed. Their young age and lean body type (low BMI) often lead to misclassification as T1DM, resulting in the use of standard insulin regimens [7]. However, T5DM patients maintain peripheral insulin sensitivity, creating a paradox. High‐dose insulin greatly increases the risk of fatal iatrogenic hypoglycemia. If misclassified as T2DM, they often do not respond to oral antidiabetics, leading to poor glycaemic control [8]. This ongoing misclassification lowers patient quality of life and increases the economic burden on LMIC healthcare systems [1].

The primary objective of this review is to provide a comprehensive synthesis of the burgeoning literature on T5DM following the IDF’s landmark 2025 decision. While covering a broad spectrum from molecular pathophysiology to diagnostic algorithms, this study specifically emphasises the pathological impact of malnutrition on beta‐cell function and the critical role of nutritional rehabilitation strategies in clinical management. Furthermore, by addressing the ongoing academic debate over whether T5DM represents a distinct disease entity or a phenotypic variant, this work aims to provide a strategic roadmap for future research.

## 2 Historical Perspective: The Evolution From J‐Type to Type 5 Diabetes

While T5DM may appear to be a contemporary addition to the literature, the links between malnutrition and glucose metabolism have been debated for nearly 70 years. Over this time, the clinical entity has been labelled with various names, depending on the region, proposed causes, and clinical findings. The first mention of malnutrition‐related diabetes in modern literature appeared in Hugh–Jones’s 1955 Jamaican observations. He described young, underweight patients with severe hyperglycemia who resisted ketosis even without insulin, a condition he termed ‘J‐type diabetes’ (Jamaica type) [9, 10]. Later, terms like ‘M‐type’ (Malnutrition‐related) in India and ‘Tropical Diabetes’ or ‘Ketosis‐Resistant Youth‐Onset Diabetes’ (KRYOD) inplaces like Nigeria emerged [11]. Early classifications focused mainly on where it occurred and on obvious clinical features, not on molecular causes.

A pivotal turning point in diabetes taxonomy came with the 1985 World Health Organization (WHO) report. It recognised malnutrition as a main cause of diabetes and introduced ‘Malnutrition‐Related Diabetes Mellitus’ (MRDM) as a third category. MRDM was divided into two subtypes: Fibrocalcific Pancreatic Diabetes (FCPD), with pancreatic calcification and exocrine insufficiency, and Protein‐Deficiency Pancreatic Diabetes (PDPD), found in those with severe protein‐energy malnutrition [12].

Despite its 1985 recognition, MRDM remained doubted in Western medical literature. In 1999, the WHO Expert Committee said that the evidence directly linking malnutrition to diabetes was insufficient. They suggested that lean diabetes may just be an unusual type of T1DM or T2DM. MRDM was downgraded to the ‘other specific types’ category, which led to less academic attention [13].

**FIGURE 1:**
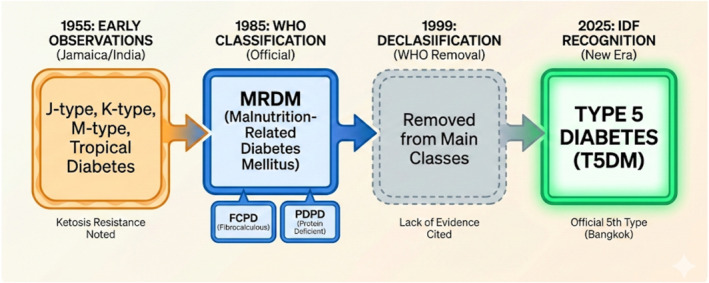
The chronological trajectory of the classification of malnutrition‐associated diabetes (1955–2025).

Following a 25‐year ‘taxonomic hiatus’, advances in precision medicine and LMIC data have revived the discussion. On 8 April 2025, at the World Diabetes Congress in Bangkok, the IDF reinstated MRDM as ‘Type 5 Diabetes’ [3]. This landmark move recognises T5DM as a distinct clinical entity with unique pathophysiological mechanisms, rather than merely a variant in the ‘other’ category (Figure 1 will come here).

## 3 Clinical Definition and Pathognomonic Features of T5DM

T5DM is mainly defined as a non‐autoimmune, ketosis‐resistant diabetes seen in young adults (< 30 years) who had chronic undernutrition in childhood or adolescence and now have a low body mass index (BMI < 18.5 kg/m^2^). T5DM is primarily characterised by a low body mass index, typically aligned with the World Health Organization (WHO) threshold for underweight of < 18.5 kg/m^2^. However, while this standardised cut‐off provides a vital universal benchmark, it is essential to consider that BMI values are ethnic‐specific and may not capture the full extent of nutritional risk in all populations. Consequently, to prevent misclassification, this fixed threshold should be utilised as part of a broader diagnostic framework that integrates the GLIM (Global Leadership Initiative on Malnutrition) criteria, which account for regional anthropometric variations and prioritise evidence of ‘life‐course undernutrition’ over isolated BMI measurements [6, 10]. The main clinical feature is its unusual metabolic profile. Even with a severe defect in pancreatic insulin secretion (low C‐peptide), patients have normal or increased peripheral and hepatic insulin sensitivity [2]. Most importantly, these patients resist ketoacidosis even after stopping insulin therapy. This remains the key distinguishing feature of T5DM [14].

Modern clustering analyses closely link T5DM with the ‘Severe Insulin‐Deficient Diabetes’ (SIDD) cluster. However, T5DM diverges from SIDD in two key ways. First, while SIDD typically affects individuals with a normal or high BMI, T5DM exclusively affects lean populations [11]. Second, while both phenotypes are non‐autoimmune, the insulin deficiency in T5DM is specifically driven by developmental beta‐cell insufficiency and malnutrition‐induced atrophy resulting from early‐life nutritional deprivation [2, 15]. This distinguishes T5DM from the classic SIDD cluster, where the deficiency is not rooted in life‐course undernutrition, and further differentiates it from the Severe Autoimmune Diabetes (SAID) cluster, which is defined by autoimmune beta‐cell destruction.

The main scholarly debate in the T5DM literature concerns how malnutrition is defined. Critics argue that defining malnutrition only by low BMI (< 18.5 kg/m^2^) is reductionist. Misra et al. call this approach ‘circular logic’. They say it fails to clarify whether leanness causes diabetes or is a result of uncontrolled hyperglycemia [16].

To resolve this diagnostic ambiguity and effectively differentiate T5DM from ‘Lean T2DM’ phenotypes, clinical evaluation must transcend isolated anthropometric measurements [17]. The T5DM framework emphasises ‘life‐course undernutrition’, the cumulative nutritional insults across an individual’s lifespan, rather than cross‐sectional thinness [8]. Consequently, integrating validated tools such as the Subjective Global Assessment (SGA) or the GLIM (Global Leadership Initiative on Malnutrition) criteria is imperative for diagnostic precision.

**FIGURE 2:**
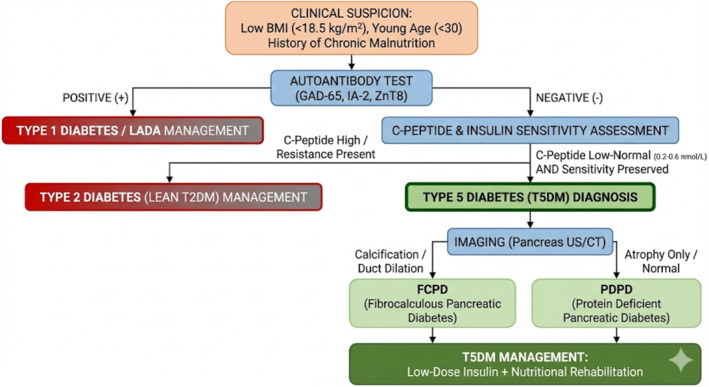
Proposed diagnostic algorithm and clinical management pathway for T5DM.

The GLIM framework mandates the presence of both ‘phenotypic’ components (low BMI, unintentional weight loss, or reduced muscle mass) and ‘etiological’ components (inadequate nutritional intake, malabsorption, or chronic inflammation) [17]. Validating T5DM as a distinct clinical entity requires multidimensional evidence of malnutrition while rigorously excluding confounding factors that contribute to wasting, such as exocrine pancreatic insufficiency, tobacco use, or chronic infection [10] (Figure 2 will come here).

## 4 Epidemiology and the Global Burden of Disease

Type 5 Diabetes Mellitus is increasingly recognised as a ‘neglected epidemic’ within the global landscape of diabetes, intrinsically linked to the socioeconomic disparities prevalent in LMICs. Recent epidemiological projections estimate that approximately 20–25 million individuals worldwide are affected by this phenotype, potentially accounting for 4%–5% of the total global diabetes burden [18]. The geographic distribution of T5DM mirrors the ‘poverty belt’, where chronic food insecurity and early‐life malnutrition remain endemic [2, 5]. Demographically, T5DM predominantly affects young adults aged 15–30 who reside in rural areas and belong to the lowest socioeconomic strata. Consequently, T5DM transcends the boundaries of a simple metabolic disorder, serving as a ‘social disease’ and a metabolic manifestation of systemic poverty and nutritional deprivation [1].

**FIGURE 3:**
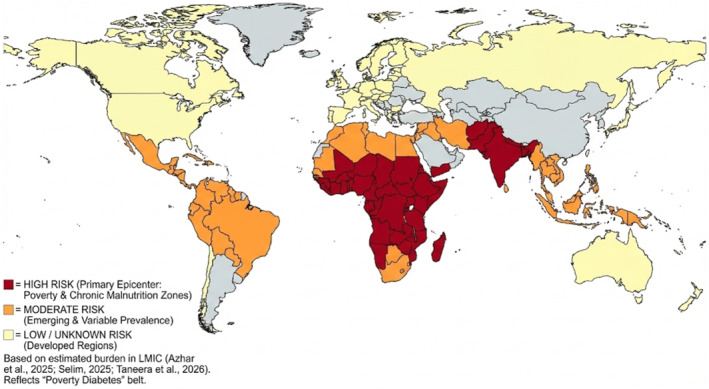
Estimated global risk map of malnutrition‐related diabetes (T5DM) illustrating the ‘Poverty Belt’ distribution.

A comprehensive analysis of the NFHS‐5 data in India identified a 0.19% prevalence of diabetes among individuals with low BMI in the 15–49 age group. Based on these findings, the actual burden of T5DM in India, including undiagnosed cases, is projected to be approximately 2.42 million, a figure that challenges previous estimates of 6 million. This discrepancy highlights how T5DM prevalence rates can fluctuate significantly based on regional screening methodologies and diagnostic precision. Recent demographic profiling reveals a significantly higher prevalence among males than among females (0.25% vs. 0.18%). Furthermore, the observation that 54% of cases are clustered within the poorest wealth quintiles reinforces the strong nutritional and economic determination of the disease [19] (Figure 3 will come here).

## 5 The Phenotypic Architecture of T5DM: Pathognomonic Findings and Differential Characterisation

T5DM exhibits a unique phenotypic architecture that is distinctly demarcated from traditional diabetes classifications. Its natural history serves as a metabolic mirror of early‐life nutritional insults manifesting in adulthood. Patients typically present before the age of 30 and are characterised by severe leanness (BMI < 18.5 kg/m^2^) and evident sarcopenia [10].

The most radical laboratory hallmark of this entity is ‘Ketosis Resistance’; patients remain resistant to ketoacidosis even upon cessation of insulin therapy, despite enduring moderate to severe hyperglycemia. Furthermore, the absence of diabetes‐specific autoantibodies (GAD‐65, IA‐2, ZnT8) and the paradoxical preservation of peripheral insulin sensitivity despite profound insulinopenia constitute the diagnostic signature of T5DM [14].

This clinical spectrum is further differentiated based on the morphological severity of pancreatic damage. FCPD represents the exocrine‐involved variant, characterised by abdominal pain, steatorrhoea, and extensive intraductal calcifications. Conversely, PDPD emerges primarily from chronic protein‐energy malnutrition, presenting as pancreatic atrophy without calcification. Current evidence suggests that these variants are not separate entities but points along a phenotypic continuum driven by the chronicity and severity of nutritional deprivation [10].

Type 5 Diabetes presents a distinct phenotype that is sharply differentiated from other types of diabetes in terms of its clinical presentation. The natural course of the disease reflects the metabolic consequences of nutritional deficiencies in early life, as seen in adulthood (Table 1 will go here).

**TABLE 1:** Comparative analysis of T5DM with classical diabetes phenotypes.

Feature	T1DM	T2DM	T5DM
Age of onset	Juvenile/young	Usually adult	Young (< 30 years)
Anthropometry	Variable	Usually overweight/obese	Lean (BMI < 18.5)
Ketosis	Prone	Resistant	Paradoxically resistant
Autoimmunity	Positive	Negative	Negative
Insulin sensitivity	Normal	Low (resistance)	High/preserved
Pancreas morphology	Immune atrophy	Normal/steatosis	Atrophy/calcification

The heterogeneous presentation of T5DM necessitates a diagnostic approach that transcends isolated parameters, such as BMI. To standardise the clinical assessment, current evidence supports the implementation of a multidimensional scoring framework. This system integrates nutritional history, physical examination, stigmata, and radiological findings to establish a comprehensive diagnostic Weight [20] (Table 2 will go here).

**TABLE 2:** Multidimensional scoring framework for T5DM diagnosis.

Clinical and laboratory criteria	Score value
Age of onset between 10 and 30 years	1
BMI < 18.5 kg/m^2^	2
History of severe childhood malnutrition	1
Physical stigmata of malnutrition (e.g., muscle wasting)	2
Ketosis resistance despite severe hyperglycemia	3
Requirement of insulin for metabolic control	2
Presence of pancreatic calcification (radiological evidence)	3
Total score	14

*Note:* Requirement of insulin for metabolic control refers to the state of absolute insulinopenia; however, clinical administration must follow low‐dose protocols to mitigate the documented risk of iatrogenic hypoglycemia.

*Clinical Interpretation*: A total score of 10 or higher strongly supports the T5DM phenotype. This standardised framework serves as a critical decision‐support tool, particularly in resource‐limited settings where advanced molecular assays, such as C‐peptide and autoantibody panels, may be unavailable.

## 6 Molecular Pathophysiology of T5DM: Developmental Programming and Beta‐Cell Failure

The IDF’s official recognition of T5DM in April 2025 represents a critical milestone in understanding the pathophysiology of diabetes in individuals exposed to chronic malnutrition. T5DM is characterised by severe hyperglycemia and significant insulin‐secretion defects; however, it remains distinct from the ‘Severe Insulin‐Deficient Diabetes’ (SIDD) cluster, which is a subphenotype of Type 2 diabetes (T2DM). While T5DM shares the insulin‐deficient feature of SIDD, it diverges through its unique developmental trajectory of life‐course undernutrition [14]. This entity is distinct from Type 1 diabetes, which involves autoimmune destruction of pancreatic beta cells, and from T2DM, which is a highly heterogeneous condition encompassing various clusters such as MARD (Mild Age‐Related), MOD (Mild Obesity‐Related), and SIDD, which exhibit varying degrees of insulin sensitivity [2]. The foundation of T5DM lies in impaired pancreatic morphogenesis and insufficient functional beta‐cell mass, resulting from chronic nutritional deprivation spanning from the foetal period to adulthood [4].

**FIGURE 4:**
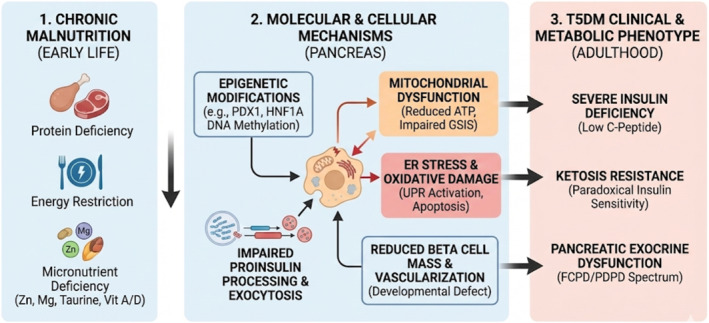
From malnutrition to beta‐cell failure: The molecular and phenotypic cycle of T5DM pathophysiology. GSIS, glucose‐stimulated insulin secretion.

The pathogenesis of T5DM is closely linked to the ‘Developmental Origins of Health and Disease’ (DOHaD) model, which posits that nutritional deficiencies during early life programme long‐term metabolic dysfunction. Energy restriction during critical growth stages permanently limits the structural development of the pancreas and the proliferative capacity of beta cells [10]. It has been observed that individuals who suffered from ‘marasmus’ (severe wasting) during childhood exhibit significantly more pronounced beta‐cell defects and glucose intolerance in adulthood compared to those who experienced ‘kwashiorkor’ (oedematous malnutrition). Epigenetic signatures and alterations in gene methylation that occur during this process suppress beta‐cell function throughout life, predisposing the individual to diabetes [6] (Figure 4 will come here).

At the subcellular level, chronic energy restriction impairs the efficiency of the mitochondrial respiratory chain, leading to diminished ATP production. This metabolic bottleneck directly inhibits Glucose‐Stimulated Insulin Secretion (GSIS), as the closure of ATP‐sensitive potassium channels is compromised. Furthermore, nutritional deprivation triggers Endoplasmic Reticulum (ER) stress by impairing protein‐folding homoeostasis, which induces the Unfolded Protein Response (UPR) and leads to beta‐cell apoptosis. In this mechanistic context, it is vital to distinguish T5DM from Maturity‐Onset Diabetes of the Young Type 5 (MODY‐5). While both phenotypes involve a reduction in functional cell mass, they arise from fundamentally different aetiologies. MODY‐5 is a primary genetic disorder caused by mutations in the HNF1B gene, typically presenting with structural anomalies such as renal cysts and pancreatic hypoplasia. In contrast, T5DM represents an acquired endocrine deficit where developmental undernutrition permanently restricts beta‐cell capacity through metabolic exhaustion rather than programmed genetic mutations [5].

**FIGURE 5:**
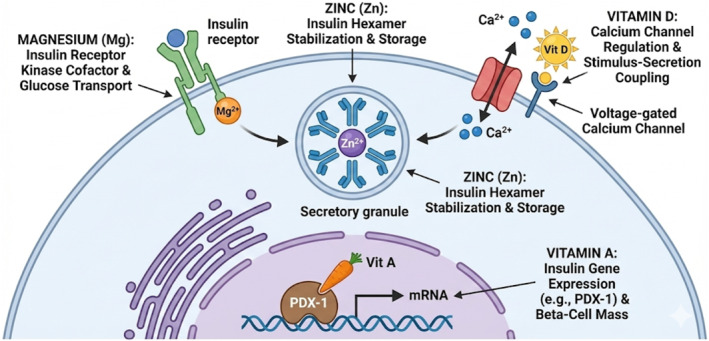
Molecular micronutrient interactions and insulin signalling pathways within pancreatic beta cells.

At the cellular level, oxidative stress induced by chronic malnutrition impairs the function of critical transcription factors, such as PDX‐1 and MafA, thereby reducing insulin gene expression [2]. However, the most striking pathophysiological hallmark of T5DM, which also serves as a critical differential marker from both T2DM and MODY‐5, is the absolute absence of ectopic fat. The metabolic distinctiveness of T5DM is further corroborated by advanced physiological assessments; specifically, euglycemic‐hyperinsulinemic clamp studies conducted in affected cohorts have consistently demonstrated high levels of peripheral and hepatic insulin sensitivity [11]. Extremely low hepatic fat fractions (e.g., 0.38%) preserve peripheral insulin sensitivity, unlike in T2DM. Conversely, micronutrient deficiencies, particularly zinc and magnesium, paralyse the cofactor systems essential for insulin synthesis, while limited glycogen and protein reserves place patients in a state of severe metabolic vulnerability. Due to this fragility, the risk of fatal ‘iatrogenic hypoglycemia’ in T5DM cases incorrectly treated with standard doses of insulin emerges as the primary clinical threat [5] (Figure 5 will come here).

## 7 Therapeutic Management of T5DM: Multidimensional Approaches and Nutritional Rehabilitation

The clinical management of T5DM requires a multidisciplinary framework that extends beyond standard glycaemic control protocols to address the systemic sequelae of chronic malnutrition. Due to the scarcity of randomised controlled trials in this field, current therapeutic strategies are largely driven by clinical consensus and observational experience, with nutritional intervention serving as the definitive cornerstone of therapy [11].

The primary strategy in T5DM management is nutritional rehabilitation, aimed at restoring body composition and pancreatic functional reserves. Due to the prevalence of severe protein‐energy malnutrition (PEM) and sarcopenia, a gradual escalation of energy intake to 35–40 kcal/kg and protein intake to 1.2–1.5 g/kg (utilising high biological value sources) is recommended. The use of locally available, energy‐dense foods is critical for the sustainability of the rehabilitation process [2]. Furthermore, the replacement of key micronutrients essential for insulin synthesis, including zinc, magnesium, and vitamins A and D, directly influences therapeutic outcomes [6] (Table 3 will go here).

**TABLE 3:** Mechanistic roles of specific nutrients in T5DM pathophysiology and clinical recommendations.

Nutrient	Mechanistic role	Clinical presentation/deficiency	Therapeutic recommendation
Protein (essential AA)	Beta‐cell vascularisation and proliferation.	Pancreatic hypoplasia, low C‐peptide.	High biological value protein (1.2–1.5 g/kg).
Taurine	Mitochondrial homoeostasis and GSIS regulation.	Increased oxidative stress, impaired insulin response.	Amino acid support or fortified diet.
Zinc (Zn)	Insulin storage and crystallisation.	Elevated proinsulin‐to‐insulin ratio.	Replacement at 100%–150% of daily requirements.
Magnesium (Mg)	Insulin receptor sensitivity and glucose transport.	Variations in insulin resistance.	Support through whole grains and oilseeds.
Vitamin D	Insulin exocytosis via calcium channel regulation.	Insufficient first‐phase insulin secretion.	2000–4000 IU/day based on serum levels.
Vitamin A	Islet development and transcriptional regulation.	Impaired foetal pancreatic development.	Balance of beta‐carotene and animal‐sourced Vit A.

Abbreviation: GSIS, Glucose‐Stimulated Insulin Secretion.

Discourse regarding insulin requirements in T5DM has been reshaped by recent clinical evidence. While high‐dose insulin was previously thought to be necessary, this requirement is now attributed to an ‘unregulated nutritional appetite’ following chronic starvation rather than true metabolic resistance. Euglycemic‐clamp studies have confirmed that T5DM patients maintain high peripheral insulin sensitivity. Consequently, meticulous management of carbohydrate distribution and appetite regulation by dietitians is vital to minimise the risk of iatrogenic hypoglycemia [21].

The pathognomonic ‘ketosis resistance’ in T5DM is not a permanent safeguard but a transient state resulting from depleted adipose tissue reserves. Following successful nutritional rehabilitation and the normalisation of energy stores, the metabolic profile may evolve towards a ‘Type 1‐like’ risk, where insulin deficiency can rapidly lead to ketosis. This underscores the importance of continuous ketosis monitoring throughout all stages of recovery [21].

While T5DM is characteristically autoantibody‐negative, clinical observations regarding immune involvement suggest a ‘limited spread of antigen reactivity’ rather than classical autoimmune destruction. This phenomenon is increasingly interpreted as a secondary metabolic inflammation (meta‐inflammation) or a localised immune response to chronic cellular stress rather than primary autoimmunity. The frequent negativity of IA‐2 antibodies and the preservation of residual C‐peptide levels further distinguish T5DM from the autoimmune‐driven beta‐cell loss seen in classic T1DM. To preserve this residual functional capacity, the integration of antioxidant micronutrients such as zinc and selenium into the dietary regimen is highly recommended [6].

## 8 Pharmacotherapy Options and Clinical Limitations

The role of oral antidiabetic agents in the management of T5DM remains a subject of ongoing debate; however, they are frequently utilised in clinical practice [14]:

- Metformin: Although T5DM is not primarily driven by insulin resistance, clinical observations have reported successful outcomes with Metformin as an ‘insulin‐sparing’ agent to improve glycaemic stability. Nevertheless, extreme caution is warranted, and its use should be avoided in patients with severe cachexia or active muscle wasting rather than using a strict BMI < 18.5 kg/m^2^ cut‐off, as the latter is a baseline characteristic of the T5DM phenotype. This nuanced approach, supported by regional observational evidence, aims to reduce the exogenous insulin requirement and the subsequent risk of iatrogenic hypoglycemia without compromising nutritional rehabilitation [14].
- Sulfonylureas: These agents may be employed to stimulate endogenous insulin secretion; however, due to the high peripheral insulin sensitivity in this phenotype, they should be initiated at minimal doses to mitigate the risk of severe hypoglycemia.
- Modern Agents: GLP‐1 receptor agonists and SGLT2 inhibitors are generally considered contraindicated in the T5DM phenotype, as they may exacerbate catabolism, trigger unintended weight loss, and worsen existing sarcopenia. (Table 4 will go here.)

**TABLE 4:** Evidence‐based intervention strategies for T5DM management.

Intervention area	Recommended strategy	Clinical rationale
Energy intake	35–40 kcal/kg/day	Rehabilitation of malnutrition and sarcopenia.
Protein	1.2–1.5 g/kg/day	Beta‐cell regeneration and muscle mass restoration.
Insulin	Low‐dose (< 0.5 U/kg)	Enhanced peripheral sensitivity and hypoglycemia prevention.
Metformin	Selective and limited use	Reported observational success, but risk of weight loss exists.
Micronutrients	Zn, Mg, Vit A, D supplementation	Essential cofactor requirements for insulin synthesis.

## 9 Comprehensive Monitoring and Complication Management

In therapeutic monitoring, HbA1c targets must be strictly individualised to minimise the risk of iatrogenic hypoglycemia [8]. The success of nutritional rehabilitation should be evaluated not only by glycaemic parameters but also by improvements in muscle strength (e.g., handgrip strength), stabilisation of BMI, and potential restoration of C‐peptide levels. Furthermore, the profound psychosocial and economic barriers faced by these patients necessitate integration with community‐based support programs and health education to enhance treatment adherence [22].

Two life‐threatening risk factors must be rigorously managed during nutritional interventions in T5DM:

- Iatrogenic Hypoglycemia: Due to the extreme peripheral sensitivity to insulin, irregularities in carbohydrate intake or inappropriate insulin dosing can lead to fatal hypoglycemic episodes. To mitigate this risk, low‐glycaemic index, complex carbohydrates (e.g., legumes, whole grains) should be evenly distributed across meals [8].
- Refeeding Syndrome: The sudden initiation of high‐calorie diets in severely malnourished T5DM patients can trigger hypophosphatemia, hypokalemia, and cardiac complications. Nutritional support should commence on 20–25 kcal/kg/day and be gradually escalated to target levels over 5–7 days [1].

As a state of severe insulin deficiency emerging within a landscape of chronic malnutrition, T5DM presents a complex prognostic profile involving both classical diabetic complications and systemic sequelae unique to nutritional deprivation. In T5DM patients, microvascular complications typically reach clinical significance within the first 5–10 years following diagnosis [2].

- Retinopathy: Current evidence indicates that the prevalence of diabetic retinopathy in T5DM is substantially high, comparable to poorly controlled T1DM cohorts.
- Nephropathy and Neuropathy: Persistent severe hyperglycemia leads to progressive renal damage and peripheral neuropathy. A significant diagnostic challenge in T5DM remains to differentiate hyperglycaemic nerve damage from nutritional neuropathies caused by vitamin (B12 and folate) deficiencies.
- Macrovascular Risk: Unlike the T2DM phenotype, conventional cardiovascular risk factors such as obesity, hypertension, and dyslipidemia are less prevalent in T5DM. However, the long‐term macrovascular consequences of vascular fragility and chronic inflammation triggered by malnutrition remain an active area of investigation [6].

T5DM acts as a systemic syndrome that alters the entire developmental trajectory of the individual, extending far beyond glucose homoeostasis [15]:

- Growth and Developmental Delay: Nutritional restriction in early life, coupled with deficiencies in insulin and Insulin‐like Growth Factor‐1 (IGF‐1), results in linear growth retardation (stunting) and significant pubertal delay.
- Bone Health and Sarcopenia: Chronic undernutrition and insulinopenia lead to reduced bone mineral density (osteopenia) and severe muscle wasting (sarcopenia), profoundly limiting physical functional capacity.
- Mortality and Survival: The prognosis of T5DM is heavily contingent upon timely diagnosis and access to appropriate insulin therapy. Fatal iatrogenic hypoglycemic episodes, often resulting from misclassification as T1DM and subsequent high‐dose insulin administration, remain the primary driver of high mortality rates in low‐resource settings.

## 10 Socioeconomic Impacts and Public Health Management of T5DM

Type 5 Diabetes transcends the boundaries of a simple metabolic disorder, serving as a biological manifestation of global poverty, food insecurity, and health inequity. Consequently, addressing T5DM necessitates a multidisciplinary approach that integrates clinical interventions with structural social reforms and community‐based educational strategies.

In the literature, T5DM is often categorised as ‘Poverty Diabetes’ or ‘Hunger Diabetes’ due to its high prevalence among the lowest socioeconomic strata [1, 5]. This conceptualisation challenges the common perception of diabetes as a ‘lifestyle disease’ of affluent societies (e.g., T2DM); instead, it highlights T5DM as the metabolic consequence of chronic nutritional deprivation and inadequate maternal‐child healthcare services. The official recognition of T5DM aligns directly with the United Nations 2030 Sustainable Development Goals (SDGs), particularly the ‘Zero Hunger’ objective [7].

The misclassification of T5DM cases as either T1DM or T2DM imposes two critical burdens on public health systems:

- Clinical Safety and Mortality: Administering high‐dose insulin based on standard T1DM protocols to highly insulin‐sensitive patients leads to fatal iatrogenic hypoglycemia, particularly in regions where food security is compromised [6].
- Economic Burden: The unnecessary utilisation of expensive insulin analogues or the prescription of ineffective oral agents due to misdiagnosis wastes limited healthcare resources in LMICs and creates a devastating financial strain on patient families [1].

Enhancing social awareness is pivotal for the early detection and management of T5DM. The ‘Type 5 Diabetes Knowledge Scale’ (T5DKS), developed by Romadlon et al., is the first validated tool designed to assess knowledge regarding the symptoms and risk factors of the disease in rural communities. Research using this scale found that approximately 78% of rural populations had never received professional diabetes education and had significant difficulty distinguishing T5DM from other diabetes types [22].

These findings underscore the urgent need for tailored educational programs that leverage locally available, protein‐rich foods (e.g., lentils, legumes) and respect cultural dietary habits. Training rural healthcare workers to recognise that ‘severe diabetes can manifest in lean individuals’ is the most strategic public health intervention to minimise diagnostic delays.

## 11 Scientific Debates and Gaps in the Literature

The official recognition of T5DM as a distinct category has not been met with universal consensus in the literature. Some researchers emphasise that the historical foundations of the October 2025 consensus statement are weak and the pathophysiological evidence remains insufficient. Specifically, using low BMI (< 23 kg/m^2^) as a surrogate marker for malnutrition is considered methodologically flawed; critics suggest this phenotype may simply be an early‐onset, insulin‐deficient subtype of Type 2 Diabetes (Lean T2DM) [16]. Basu and Maheshwari challenge the scientific validity of this classification, arguing that many cases labelled as T5DM are actually a conflation with ‘Lean T2D’ [19].

In response to these critiques, Hawkins et al. state that the T5DM definition is grounded in seven decades of data and international consensus. They confirm the existence of a unique phenotype in individuals with a BMI < 18.5 kg/m^2^ characterised by preserved insulin sensitivity despite severe secretory defects, a profile that lacks the genetic signatures of T2DM. Proponents argue that the lack of universal biomarkers should not preclude classification, as T5DM serves as a crucial ‘call to action’ for neglected populations [23].

The classification debate extends beyond theoretical taxonomy into critical clinical risk management. Pathan et al. warn that misclassifying these patients can lead to severe ‘iatrogenic harm’ in low‐income settings [8]. Limited glycogen reserves in malnourished individuals place them at a high risk of fatal hypoglycemia when treated with standard insulin doses. Thus, recognising T5DM is presented as both a scientific necessity and an ethical ‘commitment to equity’ in global health.

Conversely, Misra et al. argue that the primary obstacle is the confusion between phenotypic variation and a novel disease aetiology. They label the grouping of autoantibody‐positive and negative individuals solely based on low BMI as ‘conceptual confusion’ and emphasise that the unresolved causality, whether malnutrition is the cause or the consequence of diabetes, remains a significant hurdle [24].

Providing a broader perspective, Trimble et al. posit that the ‘Type 5 Diabetes’ proposal aims to grant clinical visibility to ‘slim’ insulin‐dependent cases in regions like Sub‐Saharan Africa (SSA). The authors argue that since the removal of the MRDM category in 1999, substantial evidence has accumulated demonstrating the profound impact of undernutrition on diabetes phenotypes [25].

From this perspective, the ‘Type 5’ classification serves less as a rigid diagnostic criterion but more as a research hypothesis designed to examine these cases through modern methodologies. It highlights that non‐obesity‐related diabetes in rural populations is shaped by unique drivers, including chronic infections, epigenetic modifications, and intergenerational nutritional deficits. Furthermore, while T5DM is predominantly linked to systemic food insecurity, its underlying pathophysiology suggests potential relevance in High‐Income Countries (HICs). Clinical malnutrition arising from early‐onset malabsorptive disorders or eating disorders may mirror these developmental insults. Moreover, recent experimental evidence indicates that voluntary caloric restriction, such as chronic intermittent fasting during adolescence, can impair β‐cell maturation and function, further suggesting that T5DM‐like phenotypes may emerge in HICs as a result of clinical or voluntary malnutrition [26].

A significant terminological conflict exists with MODY‐5. Nurkolis et al. highlight that ‘Type 5’ is already established for Maturity‐Onset Diabetes of the Young Type 5 caused by HNF1B mutations. Unlike malnutrition‐related T5DM, MODY‐5 presents with multisystemic involvement, including renal cysts and urogenital abnormalities. Therefore, renal involvement and specific genetic backgrounds must be rigorously excluded before confirming a T5DM diagnosis [27].

The formal recognition of T5DM has initiated a new era of research aimed at elucidating this neglected phenomenon at molecular and epidemiological levels; however, critical gaps remain to be addressed. Future research should prioritise the use of omics technologies to identify universal biomarkers and unique epigenetic signatures inherited from the foetal period, which are essential for distinguishing T5DM from classical diabetes types and ensuring diagnostic precision [2, 23].

Furthermore, to address the heterogeneity in malnutrition definitions, it is imperative to move beyond a sole focus on low BMI values and integrate standardised assessment tools, such as the GLIM criteria, into diabetes phenotypic characterisation. Conducting large‐scale prospective cohort studies in regions such as Sub‐Saharan Africa and South Asia remains essential for establishing the molecular causality between malnutrition and diabetes [17]. Finally, the implementation of randomised controlled trials (RCTs) to evaluate the efficacy of integrated nutritional rehabilitation combining specific amino acid and micronutrient replacements with low‐dose insulin regimens must constitute the core research agenda to develop evidence‐based therapeutic guidelines tailored to this population [10].

## 12 Conclusion

The inclusion of T5DM in the IDF’s official classification in 2025 represents a historic milestone in recognising the metabolic imprints of poverty and malnutrition in diabetology. Rather than resulting from autoimmunity (Type 1) or classic insulin resistance (Type 2), T5DM is a pathophysiologically unique entity arising from early‐life nutritional deficiencies that permanently impair pancreatic development.

This review demonstrates that T5DM is not merely a biological disorder but a clinical reflection of global food insecurity. Misclassifications as ‘Type 1’ or ‘Type 2’ in low‐income populations lead to fatal consequences, such as iatrogenic hypoglycemia, and the depletion of limited healthcare resources. From a nutrition and dietetics perspective, patient management must transcend simple glycaemic control and place ‘Nutritional Rehabilitation incorporating high‐quality protein and specific micronutrient replacements for pancreatic reserve restoration at the centre of therapy. Ultimately, further methodologically rigorous studies are required for the universal registration of T5DM by the World Health Organization (WHO). Such recognition is an ethical imperative to grant clinical visibility to this patient group, the carriers of the ‘metabolic legacy of hunger’, and to ensure global health equity.

## Author Contributions

İrem Nur Şahin Anılgan and Aslıhan Atar‐Uğurlu conceived and critically reviewed and edited the manuscript. All authors have read and approved the final manuscript.

## Funding

The authors have nothing to report.

## Ethics Statement

The authors have nothing to report.

## Conflicts of Interest

The authors declare no conflicts of interest. The figures were created using Gemini (Google AI), no copyright permission was required.

## Data Availability

No datasets were generated or analysed during the current study.
